# School learning modes during the COVID-19 response and pre- to during pandemic mental health changes in a prospective cohort of Canadian adolescents

**DOI:** 10.1007/s00127-023-02557-2

**Published:** 2023-09-05

**Authors:** Karen A. Patte, Katelyn Battista, Mark A. Ferro, Richard E. Bélanger, Terrance J. Wade, Guy Faulkner, William Pickett, Negin A. Riazi, Valerie Michaelson, Sarah Carsley, Scott T. Leatherdale

**Affiliations:** 1 Faculty of Applied Health Sciences, Department of Health Sciences, Brock University, Niagara Region, 1812 Sir Isaac Brock Way, St. Catharines, ON L2S 3A1 Canada; 2 School of Public Health Sciences, University of Waterloo, 200 University Ave, Waterloo, ON N2L 3G1 Canada; 3 Projet COMPASS-Québec, VITAM–Centre de recherche en santé durable de l’Université Laval, 2480 Chemin de La Canardière, Quebec City, QC G1J 2G1 Canada; 4 Faculty of Medecine, Departement of Pediatrics, Université Laval, Ferdinand Vandry Pavillon, 1050 Avenue de La Médecine, Quebec City, QC G1V 0A6 Canada; 5 School of Kinesiology, University of British Columbia, Lower Mall Research Station Room 337, 2259 Lower Mall, Vancouver, BC V6T 1Z3 Canada; 6 Public Health Ontario, 661 University Avenue, Suite 1701, Toronto, ON M5G 1M1 Canada

**Keywords:** Online learning, School closures, Youth, Mental health, Prospective, COVID-19

## Abstract

**Purpose:**

Considerable debate centered on the impact of school closures and shifts to virtual learning on adolescent mental health during the COVID-19 pandemic. We evaluated whether mental health changes differed by school learning modes during the pandemic response among Canadian adolescents and whether associations varied by gender and perceived home life.

**Methods:**

We used prospective survey data from 7270 adolescents attending 41 Canadian secondary schools. Conditional change linear mixed effects models were used to examine learning mode (virtual optional, virtual mandated, in-person, and blended) as a predictor of change in mental health scores (depression [Centre for Epidemiologic Studies – Depression], anxiety [Generalized Anxiety Disorder-7], and psychosocial well-being [Flourishing scale]), adjusting for baseline mental health and covariates. Gender and home life happiness were tested as moderators. Least square means were calculated across interaction groups.

**Results:**

Students learning in a blended learning mode had greater anxiety increases relative to their peers in other learning modes. Females learning fully in-person and males learning virtually when optional reported less of an increase in depression scores relative to their gender counterparts in other learning modes. Learning virtually when optional was associated with greater declines in psychosocial well-being in students without happy home lives relative to other learning modes.

**Conclusion:**

Findings demonstrate the importance of considering gender and home environments as determinants of mental health over the pandemic response and when considering alternative learning modes. Further research is advised before implementing virtual and blended learning modes. Potential risks and benefits must be weighed in the context of a pandemic.

**Supplementary Information:**

The online version contains supplementary material available at 10.1007/s00127-023-02557-2.

## Introduction

The coronavirus disease 2019 (COVID-19) pandemic and public health measures are believed to have exacerbated pre-existing concerns and inequities in youth mental health [1–9]. Since March 2020, youth experienced drastic disruptions to their lives, including the widest reaching and longest school closures in history [2]. The potential unintended consequences of school closures on adolescent mental health led to widespread concerns and public debate, with some arguing that the risks outweighed any benefits for reduced transmission [2, 8, 9, 12]. Indeed, schools provide important structure and routine, and opportunities to socialize with peers, for adult mentorship, and to engage in extracurricular and physical activities—all factors with known links to positive youth mental health [2, 3, 6–11]. Moreover, schools are often where mental health concerns are first recognized; they are key contexts for the delivery of public health interventions and the most common setting for the provision of mental health services [13–15]. Acknowledging these factors, opposing parties maintain that greater illness and death due to COVID-19 transmission in schools would have been more detrimental to young people’s mental health than temporary and partial closures [16]. Furthermore, school can also contribute to distress for some students (e.g., related to bullying and academic pressures) [17, 18].

Literature generally indicates adverse associations between remote learning and mental health relative to in-person learning during the COVID-19 pandemic [19, 20]. However, previous research largely relies on cross-sectional designs and retrospective accounts [5, 20, 22]. Where prospective evidence does exist, it typically lacks baseline pre-pandemic data and robust measures of mental health and psychopathology [5, 20, 22, 23]. Moreover, most published literature applies to the first COVID-19 wave and complete school closures; there is a need for research on the prolonged pandemic response, examining partial school closures and resultant diverse learning modes [20, 21]. Further, research on virtual remote learning needs to consider variations by gender and students’ perceptions of their home environments. Schools can offer reprieve from difficult home lives, and financial and family distress increased during the COVID-19 pandemic [1, 3, 24, 25]. COVID-19 and previous pandemics have also been shown to have greater impacts on girls/women, given differences in mental health (e.g., how problems are exhibited and managed), social relationships, risk of exposure to violence/abuse, and gendered delegation of sibling caregiving and household chores [26–28].

Using data from an ongoing Canadian prospective cohort, the objective of this study was to test whether learning modes during the COVID-19 response were differentially associated with mental health changes from before to during the pandemic in adolescents. Results may help inform school protocols and policies to support youth mental health in the case of future events. We also tested whether learning mode associations with mental health changes were modified by gender or perceived home life. We hypothesized that experiences of learning from home would vary based on whether it was mandated or optional and that virtual learning would have more adverse associations with mental health among females and non-binary youth, and adolescents without happy home lives, than their counterparts identifying as male and with positive perceptions of their home life happiness.

## Methods

### Design and sample

The *Cannabis, Obesity, Mental health, Physical activity, Alcohol, Smoking, and Sedentary behavior* (*COMPASS*) *Study* is an ongoing prospective study designed to collect hierarchical survey data annually from a rolling cohort of students in grades 9 through 12 (Secondary I–V in Quebec) and their schools [29]. School boards and schools were purposefully selected based on whether they permitted active-information passive-consent parental permission protocols, which are shown to better reach students at risk of depression [30]. All students attending participating schools and not withdrawn by their parents were eligible to participate. Students could withdraw anytime. Participant codes were generated to enable individual-level data to be linked across waves [29]. Pre-pandemic student data were collected using paper-based surveys completed during one classroom period, with an average response rate of 83.4%. Since March 2020, when schools first closed due to the COVID-19 pandemic, surveys were conducted online using Qualtrics XM survey software, with an average response rate of 58.0%. A survey link was emailed to all students by their schools, followed by a reminder email one week after [31]. Additional details regarding COMPASS study methods can be found online (www.compass.uwaterloo.ca) or in print [29], including online requests for the full student questionnaires. All procedures received ethics approval from the University of Waterloo (ORE#30118), Brock University (REB#18–099), CIUSSS de la Capitale-Nationale–Université Laval (#MP-13-2017-1264), and participating school boards.

We used 2-year linked COMPASS data from 8274 students attending 41 secondary schools that participated before the onset of the COVID-19 pandemic response (October 2019–February 2020; time 1), when all schools were open to in-person learning, and during the COVID-19 pandemic in the 2020–2021 school year (December 2020–May 2021; time 2). At baseline, 23,269 students participated in the 41 schools that participated in both study waves. Students with missing values on learning mode or covariates (*n* = 562) or on any of the three outcomes (*n* = 442) were removed, leaving a final analytic sample of 7270 students. An attrition analysis compared baseline characteristics of the full baseline sample with the final analytic sample. See Table 1 for pooled t-test and Cohen’s D results for mean differences in continuous outcomes and Chi-square tests and Cramer’s V results for proportion differences in categorical variables. The full baseline sample had poorer mental health scores, and the analytic sample were more likely to identify as female and white, be in earlier grades, and perceive a happy home life. However, the Cohen’s D and Cramer’s V results indicate a small magnitude of difference, with the exception of grade, as expected in a rolling cohort design; many non-linked students were in the final year of secondary school and would have graduated out of the cohort.

**Table 1:** Attrition analysis comparing baseline measures of the final analytic sample to the full baseline sample

	Analytic sample		Baseline sample		Chi-square		
	*n*	%	*n*	%	Value	*p* value	Cramer’s V
Total	7270	100%	23,972	100%			
*Gender*							
Female	4108	57%	11,465	48%	154.60	< .0001	0.07
Male	3034	42%	11,546	48%			
Different/Prefer Not to Say	128	2%	666	3%			
Missing	0	0%	295	1%			
*Grade*							
7/Sec 1	1106	15%	2631	11%	1387.12	< .0001	0.21
8/Sec 2	935	13%	2490	10%			
9/ Sec 3	2228	31%	5214	22%			
10/Sec 4	1959	27%	5259	22%			
11/Sec 5	972	13%	4905	20%			
12/	42	1%	2995	12%			
Other	6	0%	264	1%			
Missing	22	0%	214	1%			
*Race/Ethnicity*							
Asian	611	8%	2619	11%	162.01	< .0001	0.07
Black	96	1%	561	2%			
Latin American	118	2%	561	2%			
Multiple	447	6%	1748	7%			
Other	331	5%	1493	6%			
White	5667	78%	16,740	70%			
Missing	0	0%	250	1%			
*Province*							
AB	272	4%	905	4%	213.67	< .0001	0.08
BC	901	12%	4613	19%			
ON	2258	31%	7570	32%			
QC	3839	53%	10,884	45%			
*Home life happiness*							
Neutral/Disagree	1277	18%	4914	20%	38.66	< .0001	0.04
Agree	5969	82%	18,510	77%			
Missing	24	0%	548	2%			

	Mean	SD	Mean	SD	Pooled *t*-test			
					*t*-value	*p* value	Pooled SD	Cohen’s D
Depression (CESD-10)	7.86	5.68	8.49	5.95	– 7.96	< .0001	5.88	0.11
Anxiety (GAD-7)	5.70	5.13	5.99	5.49	– 4.08	< .0001	5.41	0.05
Psychosocial wellbeing (FS)	32.93	5.42	32.36	5.80	8.45	< .0001	5.71	– 0.10

*CESD-10*  10-item Center for Epidemiologic Studies Depression scale Revised (CESD-10) (Andresen et al. [32]), *GAD7*  7-item generalized anxiety disorder scale (GAD-7) (Spitzer et al. [33]), *FS*  Flourishing scale (Diener et al. 2010, [34])

## Measures

### Mental health outcomes

Depressive symptoms were measured using the 10-item *Center for Epidemiologic Studies Depression scale Revised* (CESD-10) [32]. Students were asked how often they experienced symptoms of major depressive disorder (e.g., “I could not get ‘going’”; “I felt everything I did was an effort”; “I felt hopeful about the future” [reverse scored]) within the last 7 days using a 4-point Likert scale (none or less than 1 day; 1–2 days; 3–2 days; 5–7 days), with items scored 0 to 3. Two positively framed items were reverse scored. Sum scores had a plausible range from 0 to 30, with higher scores indicative of more frequent symptoms. Anxiety symptoms were measured using the 7-item *Generalized Anxiety Disorder scale (GAD-7)* [33]. Students were asked how often they experienced each symptom (e.g., “becoming easily annoyed or irritable”; “not being able to stop or control worrying”) in the last 2 weeks on a 4-point Likert scale including “Not at all”, “Several days”, “Over half the days”, and “Nearly every day” (scored 0, 1, 2, and 3, respectively). Sum scores ranged from 0 to 21, with higher scores indicating more frequent symptoms. Psychosocial well-being was assessed using Diener’s 8-item *Flourishing Scale* (FS) [34]. Students were asked to indicate their agreement with statements regarding their relationships, life purpose and satisfaction, engagement and interest with daily activities, optimism, and self-esteem and competence. The original 7-point Likert scale was reduced to 5-points (“Strongly agree” to “Strongly disagree”) by removing the “Slightly agree” and “Slightly disagree” options to be suitable for a large school-based survey [35]. Sum scores ranged from 0 to 32, with higher scores indicating higher perceived well-being [36]. All three scales have demonstrated validity in adolescents [37–42] including strict measurement invariance by gender and grade in the COMPASS study [40, 41]. Continuous scores on the CESD and GAD-7 correlate strongly with comparable measures [33, 34, 38–40, 43–46], and scores of 10 or higher on the CESD and GAD-7 have demonstrated specificity and sensitivity for detecting clinical depression and generalized anxiety or other anxiety disorders, respectively [33, 34, 47]. Scores on the Flourishing Scale correlate strongly with other psychological and social well-being measures [35, 48]. Internal consistency was α = 0.81, α = 0.89, and α = 0.90 for the CESD-10, GAD-7, and FS, respectively. For each scale score, person-mean imputation was used for students missing 1 or 2 items to minimize missing data [48, 49].

### Learning mode

Schools across Canada first closed to in-person learning mid-March 2020. They remained closed for the remainder of the 2019–20 school year and implemented emergency remote learning, which varied greatly across and within schools and boards, but was minimal for many students [50]. Schools resumed in-person learning in the fall of 2020, with staggered re-openings over September 2022 in Ontario schools [51]. Localized partial school closures were again mandated in the 2020–21 academic year in areas and at times of high COVID-19 case counts, particularly in January–February 2021, during the second COVID-19 wave and the emergence of the Omicron variant, and again in May–April 2021 [50]. During closures, schools shifted to virtual remote learning, which involved a mix of synchronous and asynchronous instruction. Outside of closures, schools across provinces typically instituted cohort or “bubble” models to reduce student contact. The blended model consisted of alternating cohorts between in-person attendance and virtual remote learning, either by alternating weeks, days (i.e., every other day in many Quebec schools), blocks of days (e.g., 3 days at school, 2 days at home, etc.), or morning/afternoons. Quadmester scheduling was common, in which the school year was divided into four terms and students took two courses per term, either alternating courses weekly or two courses/day. Alternatively, some schools implemented Octomesters, in which students took one course at a time for eight terms. Additional safety protocols were initiated in schools, such as mandatory face masks and shortened lunch periods and school hours. Parents/students could also choose to remain remote when schools were open to in-person learning [51]. Students learning remotely when optional either attended virtual schools or engaged in hybrid learning, meaning educators taught students learning in-person and virtually at the same time and in the same classroom.

In 2020/21, student surveys asked what form of curriculum delivery they were currently participating in: “100% in-person (I physically go to school every day)”, “100% online (I attend school remotely over the internet every day)”, and “Alternating in-person and online (Some days I go to school, some days I attend online)”, hereby referred to as Blended. Given potential self-selection into learning mode, students in schools that were mandated to be closed to in-person learning at the time of data collection were categorized as “Virtual mandated”. Remaining students who selected 100% online were categorized as “Virtual optional”, meaning that they or their guardian(s) had opted for virtual learning when schools were open [52].

### Covariates

Models controlled for student-reported gender (male; female; “I describe my gender differently”/”I prefer not to say” [collapsed due to small sample size]), grade-level at follow-up (Grade 9 to 12 in Ontario, Alberta, and BC; Secondary 2 to 5 in Quebec, classified as equivalent to Grade 8 to 11), race/ancestry (Asian, Black, Latin American/Hispanic, Multiple [selected more than one response], Other [selected ‘other’ or Métis, First Nations, or Inuit; ethics restrictions precluded the identification of students with Indigenous heritage for separate study], White), household size including themselves, province, home life happiness (agreement with statement: “I have a happy home life”; responses dichotomized as Strongly Agree/Agree vs. Neutral/Disagree/Strongly Disagree), and socioeconomic status (SES). The home life happiness single item was derived from the Canadian Health Behaviour of School-aged Children (HBSC) study and added to the COMPASS student survey as a pragmatic measure to assess the home context, based on the need for brevity in a large school-based youth survey with multiple objectives, and the constraints of passive consent protocols. While passive consent protocols are advantageous in improving response rates, limiting selection bias, and reducing burden on schools, they prohibit the inclusion of items that may require identification of students (e.g., questions regarding experiences of abuse) [53]. An exploratory analysis of home life happiness in the HBSC study found associations with higher perceived health, fewer subjective health complaints, and lower depression and engagement in risky behaviors [54]. Adolescents that reported a happy home life were more likely to not live in a foster home and to eat meals as a family and have higher family support [54]. SES scores ranged from 0 to 8, with higher scores indicating lower SES, derived from six questions: “About how much money do you usually get each week to spend on yourself or to save?” (Zero-$5 = 2; $6-$100 = 1; > $100 = 0); “In your house, do you have your own bedroom?” (Yes = 0; No = 1); “Do you sometimes go to bed hungry because there is not enough money to buy food?” (Yes = 1; No = 0); “Would you say that you and your family are more or less financially comfortable than the average student in your class?” (More comfortable = 0; As comfortable = 1; Less comfortable = 2); “How true are the following statements about COVID-19 for you right now? I am worried about my family being able to pay bills and expenses” (True/Mostly True = 1; Neutral/I don’t know/Mostly False/False = 0); and “If you do not eat breakfast every day, why do you skip breakfast? There is nothing to eat at home” (Yes = 1; No = 0). SES responses were scaled for students missing 1 or 2 items; students missing 3 or more items were set to missing. While school data collection dates are kept similar each year, at an individual level, the number of days between data collection dates was subtracted from 365 days and entered into models to control for any differences in time between data collection dates among participants.

### Statistical analysis

Average baseline, follow-up, and within-person change in scores were calculated for CESD-10, GAD-7, and FS by each covariate. Conditional change models (i.e., lagged dependent variable models) were used with time 2 CESD-10, GAD-7, and FS scores as the outcomes to examine learning mode as a predictor of change in mental health score. Linear mixed effects regression models with random intercept for school were used to account for the clustering survey design of students within schools. All models controlled for gender, grade, race/ancestry, household size, home life happiness, SES score, province, and difference from 365 days between data collection dates. Additionally, baseline mental health scores were included as a confounding predictor of change scores [54, 55]. Learning mode interactions with gender and perceived home life happiness were tested based on hypothesized moderation effects and statistical significance of main effects of these two covariates. Note that no main effects were found for household size or race/ethnicity. Least squares means estimates for student learning mode were calculated to allow comparison of adjusted changes in mental health outcomes across learning mode interaction groups. SAS 9.4 was used for all analyses and PROC GLIMMIX was used for the linear mixed effects models.

## Results

### Descriptive statistics

Table 2 presents descriptive statistics of the sample. Over half (56.5%) of the sample identified as female, while 1.8% reported describing their gender differently or preferred not to say. Most participants identified as White (78.0%) and attended schools in Quebec (52.8%) or Ontario (31.1%). About 80% perceived having a happy home life. Students were distributed across the four learning mode categories; virtual mandated was the most common mode (28.5%), blended was the least common (22.3%), and in-person (25.2%) and virtual optional (24.0%) modes each accounted for about one-quarter of the sample.

**Table 2:** Sample descriptive statistics and mental health outcomes among Canadian adolescents before and during the pandemic response

	Sample		CESD-10			GAD-7			FS		
	n/M	%/SD	T1	T2	Delta	T1	T2	Delta	T1	T2	Delta
Total	7270	100.0%	7.9	9.8	1.9	5.7	7.1	1.4	32.9	31.8	– 1.1
*Learning mode*											
In-person	1834	25.2%	8.9	10.2	1.3	6.4	7.7	1.3	31.7	30.7	– 1.0
Blended	1619	22.3%	8.4	10.2	1.8	6.2	7.8	1.6	32.4	31.5	– 1.0
Virtual optional	1747	24.0%	6.8	8.8	2.0	4.6	6.0	1.4	34.1	33.0	– 1.2
Virtual mandated	2070	28.5%	7.4	9.9	2.4	5.5	7.0	1.5	33.4	32.0	– 1.3
*Gender*											
Female	4108	56.5%	8.8	11.3	2.4	6.8	8.7	1.9	32.7	31.2	– 1.5
Male	3034	41.7%	6.3	7.4	1.2	4.0	4.8	0.8	33.5	32.8	– 0.6
Differently/Prefer not to say	128	1.8%	13.5	16.0	2.5	10.3	12.3	2.0	27.2	25.9	– 1.3
*Grade at follow-up*											
8/Sec 2	1134	15.6%	6.0	8.7	2.7	3.8	5.8	1.9	35.1	32.8	– 2.3
9/Sec 3	940	12.9%	6.3	9.1	2.7	4.2	6.0	1.8	34.6	32.8	– 1.8
10/Sec 4	2227	30.6%	7.9	9.9	1.9	5.8	7.3	1.6	32.7	31.5	– 1.2
11/Sec 5	1965	27.0%	8.7	10.1	1.4	6.5	7.6	1.1	32.1	31.6	– 0.5
12	1004	13.8%	9.6	10.7	1.1	7.4	8.3	0.9	31.1	30.9	– 0.3
*Race/Ancestry*											
Asian	611	8.4%	9.0	10.1	1.1	6.2	7.5	1.3	31.3	30.3	– 1.0
Black	96	1.3%	7.3	9.9	2.6	4.7	6.6	1.9	32.6	31.3	– 1.3
Latin American	118	1.6%	7.2	9.4	2.3	5.8	7.5	1.7	33.2	31.8	– 1.4
Multiple	447	6.1%	9.0	11.3	2.3	6.9	8.4	1.5	31.8	30.5	– 1.2
Other	331	4.6%	9.9	11.2	1.3	6.9	8.1	1.2	30.9	29.7	– 1.3
White	5667	78.0%	7.6	9.5	2.0	5.5	6.9	1.4	33.3	32.2	– 1.1
*Household Size (including student)*											
1 person/2 people	322	4.4%	8.9	11.1	2.3	6.1	8.0	1.8	31.4	30.2	– 1.2
3 people	1277	17.6%	8.6	10.2	1.6	6.3	7.7	1.4	32.3	31.4	– 0.8
4 people	3058	42.1%	7.5	9.4	1.9	5.4	6.7	1.4	33.3	32.1	– 1.2
5 people	1682	23.1%	7.6	9.6	2.0	5.6	7.1	1.6	33.2	32.1	– 1.2
6 or more people	931	12.8%	8.2	10.2	2.0	6.0	7.4	1.4	32.5	31.3	– 1.2
*Province*											
AB	272	3.7%	9.9	11.1	1.2	7.4	8.8	1.3	31.6	30.8	– 0.8
BC	901	12.4%	9.2	10.2	1.0	6.7	7.9	1.2	31.3	30.4	– 1.0
ON	2258	31.1%	9.0	10.7	1.7	7.0	8.3	1.4	31.5	30.9	– 0.6
QC	3839	52.8%	6.7	9.0	2.3	4.6	6.1	1.5	34.2	32.7	– 1.5
*Happy Home Life*											
Neutral/Disagree	1507	20.7%	11.7	15.1	3.4	8.6	11.2	2.7	29.3	26.5	– 2.8
Agree	5763	79.3%	6.9	8.4	1.5	4.9	6.0	1.1	33.9	33.2	– 0.7
*SES score*											
[0:8]	1.9	1.2									
*Timing*											
days [ – 38:187]	94.2	60.2									

*CESD-10*  10-item Center for Epidemiologic Studies Depression scale Revised (CESD-10) Andresen et al. [32], *GAD7*  7-item Generalized Anxiety Disorder scale (GAD-7) Spitzer et al. [34], *FS*  Flourishing Scale Diener et al. 2010, [34]; *T1*  Pre-pandemic (October 2019–February 2020), *T2*  COVID-19 response (October 2020–June 2021), Timing = 365 days minus the number of days between T1 and T2 data collections

Table 2 also presents mean scores for the three mental health outcomes at time 1 and 2 by learning mode and all covariates. Mental health mean scores were lower in time 2 than time 1. There was less of a difference in all three mean unadjusted mental health outcome scores pre- to post-COVID-19 onset among students attending school fully in-person in comparison to the score differences among students in the other learning mode categories, while students attending virtually when mandated had relatively greater differences in depression scores across waves. Males had less of a difference across time 1 and 2 in unadjusted mental health outcomes relative to females and students that describe their gender differently or preferred not to say. Students in earlier grades had greater differences in scores relative to students in later grades. The largest differences in mental health scores across waves were among students that did not perceive their home life to be happy, in comparison to students with home life happiness and in all other subgroups examined.

### Conditional change models

Table 3 shows results of the main effects conditional change models; mental health outcomes from before to during the COVID-19 response were regressed on learning mode, controlling for the corresponding pre-pandemic mental health outcome, covariates, and school clustering. A main effect for learning mode indicated that students learning in a blended mode had a marginally greater increase in anxiety scores (Est. = 0.45, 95% CI [0.10, 0.81]) relative to students learning in-person. No main effect for learning mode resulted for depression or psychosocial wellbeing. Students that identified as female, described their gender differently or preferred not to say, and did not perceive their home life to be happy had greater declines in all three mental health outcomes from before to during the pandemic response, relative to their peers identifying as male and with happy home lives, respectively.

**Table 3:** Conditional change models for mental health changes before and during the pandemic response by learning mode in Canadian adolescents (*N* = 7270)

	CESD-10		GAD-7		FS	
	Est	95% CI	Est	95% CI	Est	95% CI
*Learning mode*						
Blended	0.39	– 0.01, 0.79	0.45	0.10, 0.81	0.08	– 0.27, 0.43
Virtual optional	0.01	– 0.50, 0.51	0.02	– 0.42, 0.46	0.42	– 0.01, 0.85
Virtual mandated	0.32	– 0.21, 0.85	– 0.06	– 0.51, 0.39	0.19	– 0.23, 0.61
In-person [ref]						
*Gender*						
Female	2.01	1.76, 2.25	2.05	1.83, 2.27	– 0.66	– 0.87, – 0.45
Differently/prefer not to say	3.24	2.34, 4.13	2.81	2.01, 3.62	– 1.94	– 2.73, – 1.14
Male [ref]						
*Happy home life*						
Neutral/disagree	3.68	3.38, 3.99	2.59	2.32, 2.86	– 3.92	– 4.20, – 3.65
Agree [ref]						

Notes: *CESD-10*  10-item Center for Epidemiologic Studies Depression scale Revised, *GAD7*  7-item Generalized Anxiety Disorder scale, *FS*  Flourishing Scale, *T1* Pre-pandemic (Oct 2019–Feb 2020); T2 = COVID-19 (Oct 2020–June 2021); All models included gender, baseline mental health score, race/ancestry, Grade at follow-up, household size, province, socioeconomic status score, and happy home life

Table 4 shows results of the conditional change models with the interaction terms added. Virtual learning when optional was associated with greater increases in depression (Est. = 1.28, 95% CI [0.60, 1.95]) and anxiety (Est. 0.79, 95% CI [0.18, 1.39]) in females, and greater declines in psychosocial wellbeing in students without home life happiness (Est. =  – 1.35, 95% CI [ – 2.07,  – 0.62]), relative to males and students with perceived happy home lives that were learning fully in-person, respectively. Virtual learning when mandated was also associated with greater increases in depression in females relative to males learning in-person (Est. = 0.75, 95% CI [0.10, 1.40]); whereas, students without home life happiness that were learning virtually when mandated had less of an expected increase in depression symptoms relative to their peers with home life happiness and learning in person (Est. =  – 1.03, 95% CI [ – 1.80,  – 0.25]).

**Table 4:** Conditional change models for mental health changes before and during the pandemic response by learning mode and interactions with gender and home life happiness in Canadian adolescents (*N* = 7270)

	CESD-10		GAD-7		FS	
	Est	95% CI	Est	95% CI	Est	95% CI
*Learning mode*						
Blended	0.24	– 0.34, 0.83	0.14	– 0.38, 0.66	0.05	– 0.46, 0.56
Virtual optional	– 0.66	– 1.30, – 0.02	– 0.35	– 0.92, 0.22	0.95	0.40, 1.50
Virtual mandated	0.10	– 0.56, 0.76	0.04	– 0.53, 0.62	0.30	– 0.24, 0.84
In-person [ref]						
*Gender*						
Female	1.40	0.92, 1.87	1.71	1.28, 2.14	– 0.45	– 0.87, – 0.03
Differently/Prefer not to say	2.71	1.26, 4.17	2.74	1.43, 4.06	– 2.44	– 3.74, – 1.14
Male [ref]						
Happy home life						
Neutral/disagree	4.05	3.50, 4.60	2.82	2.33, 3.31	– 3.68	– 4.17, -3.19
Agree [ref]						
*Interaction between learning mode and gender (ref: Male)*						
Learning mode * female						
Blended	0.34	– 0.35, 1.04	0.53	– 0.09, 1.16	0.09	– 0.53, 0.70
Virtual optional	1.28	0.60, 1.95	0.79	0.18, 1.39	– 0.54	– 1.14, 0.06
Virtual mandated	0.75	0.10, 1.40	0.10	– 0.48, 0.68	– 0.29	– 0.87, 0.29
In-person [ref]						
Learning mode * differently/prefer not to say						
Blended	0.04	– 2.19, 2.27	0.55	– 1.46, 2.56	1.23	– 0.76, 3.22
Virtual optional	1.71	– 1.02, 4.45	– 0.27	– 2.73, 2.20	– 0.46	– 2.90, 1.98
Virtual mandated	0.78	– 1.65, 3.21	– 0.44	– 2.63, 1.75	1.15	– 1.02, 3.32
In-person [ref]						
*Interaction between learning mode and happy home life (ref: Agreed)*						
Learning mode * neutral/disagree						
Blended	– 0.16	– 0.96, 0.63	0.02	– 0.69, 0.74	– 0.21	– 0.92, 0.50
Virtual optional	– 0.24	– 1.07, 0.59	– 0.30	– 1.05, 0.44	– 1.24	– 1.98, – 0.50
Virtual mandated	– 1.03	– 1.80, -0.25	– 0.66	– 1.35, 0.04	0.22	– 0.47, 0.91
In-person [ref]						

*CESD-10*  10-item Center for Epidemiologic Studies Depression scale Revised, *GAD7*  7-item Generalized Anxiety Disorder scale, *FS*  Flourishing Scale, *T1*  Pre-pandemic (Oct 2019–Feb 2020), *T2* COVID-19 (Oct 2020–June 2021), All models included gender, baseline mental health score, race/ancestry, Grade at follow-up, household size, province, socioeconomic status score, and happy home life

**Fig. 1:**
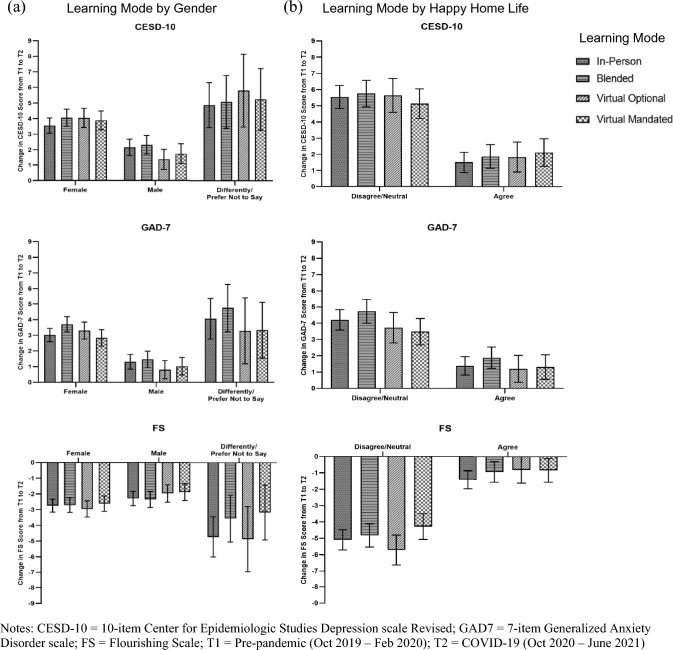
Least squares means estimates for changes in mental health outcomes from before to during the COVID-19 pandemic response in Canadian adolescents by student learning mode and gender (**a**) and home life happiness (**b**)

Least square means estimates are presented in the Supplementary Table S1. Figure 1 displays the least squares means estimates for changes in mental health outcomes by learning mode and gender (Fig. 1a) and learning mode and perceived home life happiness (Fig. 1b) before and during the COVID-19 response. Females learning fully in-person during the pandemic year had marginally less of an increase in depression scores relative to females learning in other modes, whereas males learning virtually when optional had less of an increase in depression than males in other learning modes. As above, students learning in a blended mode had greater increases in anxiety that their peers in other learning modes, regardless of gender and home life happiness. In students without happy home lives, learning virtually by choice was associated with greater declines in psychosocial wellbeing relative to other learning modes. Caution is advised in interpreting differences among students identifying their gender differently given the large confidence intervals and smaller cell sizes.

## Discussion

In a prospective cohort of Canadian adolescents, we examined whether mental health changes from pre-COVID-19 to the first full school year of the pandemic differed by school learning modes. In non-adjusted descriptive statistics, students learning fully in-person had less of a decline in mental health, and students learning in virtual mandated modes had greater depression increases, than their peers in other learning modes. However, our adjusted models demonstrated negligible differences in mental health outcomes by learning mode. In the main effects models, adjusting for baseline mental health, covariates (socioeconomic status indicators, household size, province, gender, and home life happiness), and school clustering, a learning mode effect was found for anxiety but not depression or psychosocial well-being; students learning in a blended mode had greater anxiety increases relative to their peers attending fully in-person. Interaction models supported our hypotheses that learning mode effects varied by students’ gender and perceived home lives. Smaller increases in depression were associated with learning fully in-person among females and learning virtually when optional among males relative to their gender counterparts in other learning modes. Conversely, among students without happy home lives, virtual learning when optional was associated with greater declines in psychosocial wellbeing relative to other learning modes.

Caution may be advisable when implementing blended learning modes, which had the only main effect and was consistent across gender and home life happiness for changes in anxiety. Blended modes divided students into alternating in-person and virtual learning cohorts, with patterns varying from alternating days to weeks. The constant back-and-forth in a blended learning format potentially requires continuing adjustment, which may have contributed to greater anxiety increases in these students relative to their peers attending school fully in-person. In a clinical sample of youth with physical illness and their parents, youth psychological distress did not differ by learning mode and parent psychological distress was lower in blended modes, but COVID-19-related worries were higher in youth and parents in virtual and blended modes relative to in-person learning [56]. Relatively few studies have examined blended learning modes, with most research focusing on pedagogical aspects rather than mental health outcomes, or only comparing fully remote and in-person modes. Amid projections that blended learning will become the norm beyond pandemic times, these results support the need for further research prior to expanding alternative learning modes.

Learning mode associations with changes in depression differed by gender. As hypothesized, learning fully in-person appeared beneficial among females relative to learning fully or partly virtual. Conversely, in males, learning virtually when optional was associated with smaller increases in depression than other learning modes controlling for perceived home lives, baseline mental health, and other covariates. In-person contact may be more critical for girls, given their socialized greater tendency to rely on social networks for support than boys. Further, given continued gendered roles and expectations, girls learning virtually may have been more likely to be expected to contribute to caregiving and household chores than boys. It is important to recognize that females consistently report greater internalizing symptoms, whereas externalizing symptoms are typically higher in males but were not assessed in this study [59]. It is also plausible that in-person learning was not as beneficial to males given the COVID-19 protocols, including mandatory masks, reduced breaks and lunch hours, quadmester or octomester scheduling, and reduced extracurricular and sport opportunities. Finally, the option of virtual learning, as opposed to mandated learning, may have supported their agency, an important aspect of healthy youth development. It is not clear from this research how decisions were made regarding learning mode when optional or how this time learning virtually was used. Further research is needed to explore findings regarding experiences of optional virtual learning, as opposed to mandated closures, including sustained effects on social, emotional, and physical health and development.

As hypothesized, mental health changes associated with virtual learning varied by how students perceived their home lives. Learning virtually when schools remained open was associated with relatively more adverse changes in psychosocial wellbeing in students without perceptions of a happy home life than their peers attending in other learning modes. Similarly, in the US high school students whose families had a choice of learning mode, attending remotely was associated with lower levels of social and emotional well-being than in-person learning [19]. Learning remotely prevented respite from difficult home lives, which may have been exacerbated during the COVID-19 pandemic [1, 3, 24, 25]. Family climate, conflict, and financial concerns have been associated with poorer mental health in adolescents over the pandemic [57, 58]. While we controlled for pre-pandemic mental health and socioeconomic factors, families or students choosing remote learning may have experienced greater pandemic-related distress in their household. Adolescents or their families may have opted for them to continue learning online during the pandemic if they had recently experienced, or were at greater risk of, deteriorations in their mental health. In a US study, parents’ decisions around learning modes were driven by perceptions of risk, parental availability, and access to in-person education [59]. A web survey found 9% of US adolescents reported a preference for fully online school, compared to 18% for blended learning and 65% that opted for learning fully in-person [60]. It is plausible that students without happy home lives had limited say in the decision. Beyond the home, youth voices have been absent in pandemic-related decision-making, despite their recognized right to be involved in decisions that impact them [61].

Results support the importance of differentiating between optional and mandated virtual learning in future research. Schools were mandated to close to in-person learning in response to worsening COVID-19 transmission rates in the community. For this reason, the virtual mandated group may have been expected to have experienced greater declines in mental health relative to students at schools that remained open. While unadjusted changes in mental health appeared more adverse in the mandated virtual group, no main effects resulted in adjusted models relative to in-person learning. Contrary to expectations, more adverse associations emerged for virtual or blended learning when schools remained open and not during mandated closures. Paradoxically, students without happy home lives and learning virtually when mandated reported less of an increase in depression symptoms relative to their peers with happy home lives learning in person; however, examining the least squares means suggests marginal differences in depression within students without happy home lives.

We were unable to rule out the possibility of unmeasured confounds and to demonstrate causality. Students were not randomly assigned to different learning modes; however, the prospective data allowed us to control for pre-existing baseline mental health and socioeconomic differences. As discussed above, mental health changes experienced by students, or other contributing factors (e.g., caregiving for a family member, poorer school experiences such as bullying victimization), may have influenced their or their family’s decision regarding attending in person, online, or in a blended mode, when provided the option. Comparisons of unadjusted and adjusted changes in mental health by learning mode, and differences between virtual optional and mandated modes, support the importance of considering self-selection, confounding, controlling for baseline mental and additional differences in students attending different learning modes. Changes in learning modes during the COVID-19 pandemic cannot be studied outside of broader lockdown measures [20]. While we controlled for province, variations in COVID-19 case rates and implemented protocols and policies occurred across and within provinces [50, 51].

The time between the two waves of the study used may miss changes in mental health over the dynamic pandemic response that more frequent assessments would have uncovered. Data collected between these two waves (i.e., spring 2020) were excluded due to lower response rates and higher risk of selection bias, and linkage across the additional waves would have further reduced the sample. Learning mode was assessed at the time of participation and changes in learning modes outside of data collection dates were not captured. The change from paper-and-pencil to online surveys may introduce bias. Study attrition and the relatively lower online response rates during the pandemic may also introduce selection bias; nonparticipation or non-linkage may have been more common among students with poorer mental health and experiences of new learning modes. Although attrition during the pandemic was at least partly due to random factors, such as whether schools administered the survey during class time. The attrition analysis indicated poorer mental health scores in the baseline full sample than the analytic sample. However, many non-linked students were in Grade 12/Secondary 1 and would have graduated out of the cohort; these students may partly account for baseline differences, as mental health is shown to decline with increasing grade [62–64].

Our sample is also a limitation, as the majority of students identified as White. Small sample sizes in some population subgroups, including within racialized and ethnic identity groups, required categories to be collapsed and limited our ability to examine within and between group differences in learning mode effects and intersectional identities. The number of students that identified their gender differently or preferred not to say prohibited interpretation of interaction terms for this group. The gender measure itself was also a limitation; more recent waves of the COMPASS study have an improved measure of gender identity with additional response options, and a separate item assessing sex assigned at birth. Further research is needed among gender diverse youth, given evidence of disproportionately adverse impacts of the pandemic measures [65, 66]. Remote learning may have been more detrimental, given that gender diverse youth are less likely to feel safe at home and connected to their families [67, 68], and connections with school adults are shown to be protective for adverse health outcomes [69]. That said, gender diverse youth experienced higher rates of bullying victimization than their cis-gender peers, with face-to-face school the primary context for bulling [70].

## Conclusion

Findings demonstrate the importance of differentiating whether virtual learning was optional or mandated in research, and considering gender and home environments as determinants of mental health over the pandemic response and when considering alternative learning modes. No one learning mode had beneficial associations with mental health changes over the pandemic for all students when adjusting for pre-COVID-19 mental health, sociodemographic factors, and school-level clustering. Caution and further research are advised before implementing virtual and blended learning modes. Potential risks and benefits must be weighed in the context of a pandemic or related crisis, with priority consideration for the health and safety of equity deserving populations. It is critical that youth voices are central these decisions.

### Supplementary Information

Below is the link to the electronic supplementary material.

Supplementary file1 (DOCX 21 KB)

## Data Availability

COMPASS study data are available upon request through completion and approval of an online form: https://uwaterloo.ca/compass-system/information-researchers/data-usage-application. The datasets used during the current study are available from the corresponding author on reasonable request.
