# Supplementary material for: School learning modes during the COVID-19 response and pre- to during pandemic mental health changes in a prospective cohort of Canadian adolescents
Source: Soc Psychiatry Psychiatr Epidemiol. 2023 Sep 5;59(1):137–50. doi: 10.1007/s00127-023-02557-2 (PMC10799804; doi:10.1007/s00127-023-02557-2)
Supplement: Supplementary file 1 — Supplementary file1 (DOCX 21 KB) [file 127_2023_2557_MOESM1_ESM.docx]

|  |  | **CESD-10** | | **GAD-7** | | **FS** | |
| --- | --- | --- | --- | --- | --- | --- | --- |
|  |  | Est. | 95% CI | Est. | 95% CI | Est. | 95% CI |
| *LS Means from Main Effect Models:* | |  |  |  |  |  |  |
| Learning Mode | Blended | 3.85 | 3.29, 4.41 | 3.13 | 2.64, 3.62 | -3.04 | -3.51, -2.56 |
|  | Virtual Optional | 3.47 | 2.85, 4.08 | 2.70 | 2.16, 3.24 | -2.70 | -3.22, -2.18 |
|  | Virtual Mandated | 3.78 | 3.16, 4.40 | 2.62 | 2.08, 3.16 | -2.93 | -3.44, -2.41 |
|  | In-Person | 3.46 | 2.95, 3.97 | 2.68 | 2.26, 3.12 | -3.12 | -3.55, -2.68 |
| *LS Means by Learning Mode and Gender from Full Interaction Models* | | | | | | | |
| Learning Mode * Female | Blended | 4.05 | 3.51, 4.60 | 3.70 | 3.22, 4.18 | -2.72 | -3.18, -2.25 |
|  | Virtual Optional | 4.04 | 3.43, 4.66 | 3.30 | 2.76, 3.84 | -2.96 | -3.47, -2.44 |
|  | Virtual Mandated | 3.88 | 3.28, 4.49 | 2.83 | 2.31, 3.36 | -2.62 | -3.11, -2.13 |
|  | In-Person | 3.55 | 3.06, 4.04 | 3.02 | 2.59, 3.44 | -2.75 | -3.16, -2.34 |
| Learning Mode * Differently/Prefer Not to Say | Blended | 5.07 | 3.37, 6.76 | 4.75 | 3.23, 6.27 | -3.56 | -5.06, -2.06 |
|  | Virtual Optional | 5.79 | 3.46, 8.13 | 3.28 | 1.18, 5.39 | -4.87 | -6.95, -2.79 |
|  | Virtual Mandated | 5.23 | 3.25, 7.22 | 3.32 | 1.54, 5.10 | -3.17 | -4.93, -1.42 |
|  | In-Person | 4.86 | 3.42, 6.30 | 4.05 | 2.76, 5.34 | -4.73 | -6.01, -3.45 |
| Learning Mode * Male | Blended | 2.31 | 1.72, 2.91 | 1.46 | 0.93, 1.98 | -2.35 | -2.86, -1.84 |
|  | Virtual Optional | 1.37 | 0.71, 2.02 | 0.80 | 0.23, 1.38 | -1.96 | -2.52, -1.41 |
|  | Virtual Mandated | 1.74 | 1.09, 2.39 | 1.02 | 0.46, 1.59 | -1.88 | -2.42, -1.35 |
|  | In-Person | 2.15 | 1.62, 2.68 | 1.31 | 0.84, 1.78 | -2.29 | -2.75, -1.84 |
| *Least Square Means by Learning Mode and Happy Home Life from Full Interaction Models* | | | | | | | |
| Learning Mode * Agree | Blended | 1.87 | 1.14, 2.60 | 1.88 | 1.23, 2.53 | -0.93 | -1.56, -0.30 |
|  | Virtual Optional | 1.83 | 0.91, 2.76 | 1.20 | 0.38, 2.03 | -0.80 | --1.61, 0.00 |
|  | Virtual Mandated | 2.11 | 1.25, 2.96 | 1.31 | 0.56, 2.06 | -0.83 | -1.56, -0.10 |
|  | In-Person | 1.50 | 0.86, 2.13 | 1.38 | 0.82, 1.94 | -1.42 | -1.97, -0.87 |
| Learning Mode * Disagree/Neutral | Blended | 5.76 | 4.94, 6.57 | 4.73 | 4.00, 5.45 | -4.82 | -5.53, -4.11 |
|  | Virtual Optional | 5.64 | 4.59, 6.69 | 3.72 | 2.79, 4.65 | -5.72 | -6.64, -4.80 |
|  | Virtual Mandated | 5.13 | 4.21, 6.05 | 3.48 | 2.66, 4.29 | -4.29 | -5.08, -3.50 |
|  | In-Person | 5.55 | 4.83, 6.26 | 4.20 | 3.57, 4.83 | -5.10 | -5.71, -4.48 |

Notes: CESD-10 = 10-item Center for Epidemiologic Studies Depression scale Revised; GAD7 = 7-item Generalized Anxiety Disorder scale; FS = Flourishing Scale; T1 = Pre-pandemic (Oct 2019 – Feb 2020); T2 = COVID-19 (Oct 2020 – June 2021); All models included gender, baseline mental health score, race/ancestry, Grade at follow-up, household size, province, socioeconomic status score, and happy home life.

**Supplementary Table S1.** Least Squares (LS) Means Estimates for mental health changes before and during the pandemic response by learning mode and interactions with gender and home life happiness in Canadian adolescents (N = 7270)
